# Health expenditure, child and maternal mortality nexus: a comparative global analysis

**DOI:** 10.1186/s12914-018-0167-1

**Published:** 2018-07-16

**Authors:** Rezwanul Hasan Rana, Khorshed Alam, Jeff Gow

**Affiliations:** 1 School of Commerce, University of Southern Queensland, Toowoomba, Australia; 2 School of Accounting, Economics and Finance, University of KwaZulu-Natal, Durban, South Africa

## Abstract

**Background:**

This paper provides empirical evidence on how the relationship between health expenditure and health outcomes varies across countries at different income levels.

**Method:**

Heterogeneity and cross-section dependence were controlled for in the panel data which consist of 161 countries over the period 1995–2014. Infant, under-five and maternal mortality along with life expectancy at birth were selected as health outcome measures. Cross-sectional augmented IPS unit root, panel autoregressive distributed lag, Dumitrescu-Hurlin and Toda-Yamamoto approach to Granger causality tests were used to investigate the relationship across four income groups. An impulse response function modelled the impact on health outcomes of negative shocks to health expenditure.

**Results:**

The results indicate that the health expenditure and health outcome link is stronger for low-income compared to high-income countries. Moreover, rising health expenditure can reduce child mortality but has an insignificant relationship with maternal mortality at all income levels. Lower-income countries are more at risk of adverse impact on health because of negative shocks to health expenditure. Variations in child mortality are better explained by rising health expenditure than maternal mortality. However, the estimated results showed dissimilarity when different assumptions and methods were used.

**Conclusion:**

The influence of health expenditure on health outcome varies significantly across different income levels except for maternal health. Policymakers should recognize that increasing spending has a minute potential to improve maternal health. Lastly, the results vary significantly due to income level, choice of assumptions (homogeneity, cross-section independence) and estimation techniques used. Therefore, findings of the cross-country panel studies should be interpreted with cautions.

## Background

Over the past few decades the world has seen substantial improvements in health outcomes (HO). This has coincided with rising health expenditure (HE). Global per capita HE has increased from US$587 in 2000 to US$1299 in 2015 in real terms [1]. Globally, since 1990 to 2013, the under-five mortality rate (U5MR) decreased by 49%, the reduction in maternal mortality ratio was 45% and life expectancy at birth (LFE) increased from 64 years to 71 years [2].

A large literature has examined the variations in HO and HE across countries [3–6]. Despite these efforts the causal relationship between HE and HO is still not clear. Researchers are yet to confirm whether income plays a key moderating role in deciding the direction of causality. Moreover, past empirical studies have overlooked the impact on HO due to a negative shock to HE. The question remains: how much variation in HO can be explained by HE? Equally important is to understand the effect of the assumptions of homogeneity and cross-section independence on the empirical findings of earlier studies. Lastly, wide disagreement regarding the variables which most accurately measure HO exists [7, 8].

It is usually assumed that rising HE will automatically improve HO. Nonetheless, the evidence for a causal association between the two variables remains inconclusive. Some studies have found no causal relationship or an insignificant association [6, 9]. Gupta et al. [10] sampled 50 developing and transition countries for 1993 and 1994 and concluded that increased HE reduces IMR and the under-five mortality rate (U5MR). Crémieux et al. [11] found that lower HE was associated with increased IMR and decreased LFE in selected Canadian provinces. Other panel data studies have found a significant association and concluded that HE plays an important role in improving HO [12, 13].

Other studies have found no evidence that total HE has any significant impact on HO [9, 14, 15]. Therefore, whether HE can significantly influence the different measures of HO remains unclear, and warrants further investigations.

Past studies have shown contradictory findings on the scale of association across low and high-income countries. Bradley et al. [16] and Gupta et al. [10] concluded that public HE provides a higher return to HO for poor countries than for high-income countries. Similarly, Self and Grabowski [17] found that HE has a significant impact on health only in low and middle-income countries. Furthermore, Bidani and Ravallion [18] and Nicholas et al. [19] stated that public HE is useful for the poor but not for the non-poor (high-income) in improving HO. Nicholas et al. [19] also concluded that private HE has no significant impact in reducing child and maternal mortality in 40 countries of sub-Saharan Africa. Similar results were also found by Anyanwu and Erhijakpor [20] for 47 African countries and Farag et al. [12] for 133 low and middle income countries. Contradicting these findings, Hall et al. [13] examined OCED countries, Jakovljevic et al. [21] with 24 European Union countries and Vavken et al. [22] with selected European Union countries all concluded that HE has had a significant impact on HO. Again, Bokhari et al. [23] in developing countries found a significant relationship between HE and maternal mortality rate (MMR). Nixon and Ulmann [5] indicated that HOs are significantly influenced by factors like diet, lifestyle and the environment. These differ significantly amongst high and low-income countries, subsequently, the influence of HE on health should also vary. Therefore, drawing a conclusion as to whether HE influences HO equally at all income levels is still not clear.

Noticeably, the literature suffers from several methodological shortcomings. This study will make some significant methodological contributions to overcoming that deficit. Firstly, no previous studies have used a comprehensive sample of panel data over a substantial time period and a large number of countries, representing all income levels and regions. Previous studies either used a small number of countries or smaller time periods. Again, the time series and panel analyses in earlier research assumed homogeneity and cross-section independence in the data [17, 24]. However, these assumptions are not always valid for panel data analyses. The results of these studies are not robust if they fail to account for unobserved heterogeneity [25]. Moreover, the presence of cross-section dependence in panel data can seriously compromise the stationarity of the variables and cause the regression results to be spurious [26]. This issue will be addressed by using recently developed estimation techniques (cross-sectional augmented IPS (Im-Pesaran-Shin) test and Dumitrescu-Hurlin (DH) causality test for heterogeneous panel) to overcome this problem. Lastly, no previous study has examined the impact on HO of negative shocks to HE. Impulse response function (IRF) and forecast-error variance decomposition (FEVD) tests are innovative methods for finding sources of information and transmission of information in a dynamic panel analysis [27]. Therefore, these tests will be employed to understand the responsiveness of HO to negative shocks in HE at different income levels.

The objective of this study is to examine these relationships using a comprehensive data set of 161 countries, divided into four income groups (see Appendix). By comparing the respective conclusions from each income group, it will be possible to examine the moderating role of income on the HE-HO nexus. Robustness of results will be enhanced by using new, appropriate estimation techniques at each stage. To the best of authors’ knowledge, no previous studies examined the HO and HE relationship using panel data have utilised these techniques. Lastly, an examination of which of four HO measures (IMR, U5MR, MMR and LFE) are more responsive to changes in HE at different income levels will be made.

This paper has of five sections. After this introductory part, the method and model structure, data and estimation strategy is presented in section “Methods”. Section “Results” reports the results whilst a discussion and some policy implications of these are presented in section “Discussion”. Lastly, section “Conclusions” will offer conclusions and outline some limitations of the study.

## Methods

### Model structure

The model structure has been derived from the Grossman demand for health model [28].

The Grossman model specifies the gross investment in stock of health with the following equation,

$$ {\mathrm{I}}_{\mathrm{t}}={\mathrm{I}}_{\mathrm{t}}\left({\mathrm{M}}_{\mathrm{t}},{\mathrm{TH}}_{\mathrm{t}},{\mathrm{E}}_{\mathrm{t}}\right) $$

In the above equation, M_t_,TH_t_ and E_t_ imply medical care, time input in gross investment function and stock of human capital, respectively. Any changes to these variables also changes the net investment in stock of health. Nonetheless, medical care, being the most important market good component of the gross investment function [28], has prices and costs associated with it. Therefore, holding other things constant, higher utilisation of medical care is related to higher health care expenditure (HE) and vice versa. Hence, the volume of medical inputs used is a function of the level of HE,

$$ {\mathrm{M}}_{\mathrm{t}}=f\left({\mathrm{HE}}_{\mathrm{t}}\right) $$

Therefore, the current study assumes that with the growth of per capita health expenditure, health status or outcomes also increases, significantly.

### Data

A heterogeneous panel data method for investigating the causal relationship between HE and HO is adopted. The data is annual for 161 countries for the period 1995 to 2014. The source of the data set are the World Development Indicators [1] and the Global Health Observatory [29]. HE per capita was used as the predictor variable for the cointegration tests. Four variables will be used as a proxy for measuring HO following the previous studies of Wang [30], Bokhari et al. [23] and Anyanwu and Erhijakpor [20]. The variables are infant mortality rate (IMR) per 1000 live births, under-five mortality rate (U5MR) per 1000 live births, maternal mortality ratio (MMR) per 100,000 live births and life expectancy (LFE) at birth. Due to the unavailability of reliable data, other measures of HO like ‘quality-adjusted life years’ or ‘potential years of life lost’ were not used.

Table 1 provides a brief comparison of the changes in values of the variables from 1995 to 2014. Noticeably, during these 20 years, real HE per capita increased almost three fold, LFE increased by more than seven years, IMR and U5MR reduced by half, and MMR has reduced by approximately two-fifths.

**Table 1:** Summary statistics (Global)

Year		1995	2014	1995	2014	1995	2014
Variables	Obs	Mean	Mean	Std.Dev	Std.Dev	Max	Max
HE	161	443.4	1202.5	819.8	1971.8	4308.5	9673.5
LFE	161	54.5	61.7	12.2	10.6	73.5	76.7
U5MR	161	64.5	30.9	65.2	32.6	279.5	162.2
IMR	161	43.7	23.2	37.4	21.8	153.4	98.8
MMR	160	277.9	157.8	403.1	227.2	2900	1410

Source: World Bank (2016)

Notes: *Obs* Number of observations, *Max* Maximum value and *HE* Health expenditure per capita, *LFE* Life expectancy at birth, *U5MR* Under-five mortality rate, *IMR* Infant mortality rate and *MMR* Maternal mortality rate

### Estimation strategy

The objective of this study is to extend the existing knowledge by using a heterogeneous panel data analysis instead of a homogenous approach, along with the assumption of cross-section dependence. Diagnostic tests confirmed that measures of HO (IMR, MMR, U5MR and LFE) and HE data contain heterogeneity and indicate a cross-section dependence problem.

#### Cross-section dependence and heterogeneity in panel data

Cross-section dependence generally arises when the error-terms of the adjacent units (country, company or state) are correlated, often due to spillover effects [31] or unobserved common factors [32]. Many previous studies have investigated the issue of cross-section dependence in panel data and cautioned against ignoring the problem [33]. The issue can create considerable difficulty for the unit root test [26], and may lead to inaccurate estimates [34] and biased standard errors [35]. There are several reasons (spatial correlation, distance, and common unobserved elements) which may be responsible for the dependence [36].

Panel data studies with N > T often fail to provide evidence for the homogeneity of the pooled data [37] which means that observations from the identical units tend to be much more similar compared to the observations of different units [38]. According to Hauck and Zhang [39] common omitted variables or events such as, global shocks impact each observational unit asymmetrically. The presence of heterogeneity creates a minor nuisance for inferences [31] and may lead to inconsistent estimates of the parameters [40].

#### Panel unit root tests

In the first step of the analysis, the stationarity of the data have been examined with the cross-sectional augmented IPS (CIPS) unit root tests [41]. The third generation unit root test allows heterogeneity of the autoregressive coefficients and gives consistent results in the presences of cross-section dependence in the panel data [42]. In addition, the HT (Harris-Tsavails) panel unit root test was used [43]. It was developed specifically for data sets with large (*N*) and small (*T*) with the option of controlling cross-sectional means.

#### Panel cointegration tests

In the second stage, the autoregressive distributed lag (ARDL) (mean group) technique was used to understand the cointegration relationship. According to Pesaran et al. [44], the ARDL can incorporate the heterogeneous panel into the error-correction model. The mean group (MG) estimation technique allows the long-run and short-run effects to be different and be heterogeneous across panel units [45]. The ARDL model is expressed as:

$$ \Delta \ {HO}_{it}={\varnothing}_i\ {HO}_{i,t-1}+{HE}_{i,t-1}{\beta}_i+{\sum}_{j=1}^{p-1}{\alpha}_{ij}\ \Delta  {HO}_{i,t-1}+{\sum}_{j=0}^{q-1}{\partial}_{ij}\ \Delta  {HE}_{i,t-j}+{\varepsilon}_{it}+{\cup}_{it} $$

where *HO* is the dependent variable with lag *p* and *HE* is the independent variable with *q* number of lags. *β*_*i*_ is the long-run coefficient and ∅_*i*_represents scalar coefficients on the lagged dependent variable which measures the speed of adjustment to the long-run equilibrium. ∂ is the short-run coefficient for independent variable and *α* is for the dependent variable. In addition, subscript *i* and *t* indicate the country and time indexes of the panel data set respectively. To determine the lag length, the standard lag selection criteria of Akaike information criterion (AIC) and Bayesian information criterion (BIC) were used. ARDL techniques are used to examine the cointegrating relationship among variables which are not stationary of the same order [44, 46].

Further the FMOLS (Fully modified ordinary least squares) method developed by Pedroni [47] which incorporates the semi-parametric correction of the OLS estimation suggested by Hansen and Phillips [48] is also utilised as the majority of the data are stationary at *I(1)* and the approach provides reliable estimates for small samples [49]. The cointegration system for panel data is:

$$ \kern2.25em {HO}_{it}={\beta}_0+{\mathrm{HE}}_{it}{\beta}_i+{\upvarepsilon}_{it} $$

and

$$ {HE}_{it}={\mathrm{HE}}_{i,t-1}+{\mathrm{v}}_{it} $$

where the vector error process *ϑ*_*it*_= (${\mathsf{\varepsilon}}_{it},{\mathsf{v}}_{it}\Big)$ is stationary with asymptotic covariance matrix represented by Ω_i_ . The variables, *HO*_*i*_ and *HE*_*i*_, have long-run cointegration with cointegrated vector *β*_*i*_, if the *HE*_*it*_ is integrated of *I(1)* [47]. In addition, FMOLS uses a semi-parametric correction for endogeneity and serial correlation. Moreover, the group mean estimator of FMOLS allows for a higher degree of heterogeneity to be present in the dynamics underlying dependent and independent variables [50, 51].

Based on Pedroni [47], the FMOLS estimator is:

$$ {\widehat{\beta}}_{i, FMOLS}={\mathrm{N}}^{-1}\sum \limits_{i=1}^N\ {\left(\sum \limits_{i=1}^T{\left({HE}_{it}-{\overline{HE}}_{it}\right)}^2\right)}^{-1}\left(\sum \limits_{i=1}^T\left({HE}_{it}-{\overline{HE}}_{it}\right){HO}_{it}^{\ast }-{T}_{\tau_i}\right) $$

where $$ {HO}_{it}^{\ast } $$ = $$ \left({HE}_{it}-{\overline{HE}}_{it}\right)-\left(\frac{{\widehat{\Omega}}_{21i}}{{\widehat{\Omega}}_{221}}\right)\Delta  {HE}_{it} $$ and

$$ {\widehat{\tau}}_i={\widehat{\varGamma}}_{21i}+{\widehat{\Omega}}_{21i}^0-\left(\frac{{\widehat{\Omega}}_{21i}}{{\widehat{\Omega}}_{221}}\right)\left({\widehat{\varGamma}}_{22i}+{\widehat{\Omega}}_{22i}^0\right) $$

where $\widehat{\Omega}$ represents the covariance and $\widehat{\varGamma}$ indicates the sums of autocovariance acquired from the long-run covariance matrix. In addition, ${\widehat{\tau}}_i$ is the moderator to correct for the autocorrelation which arises from the heterogeneity dynamics determining dependent and independent variables in the short run process [49].

#### Panel granger causality tests

Next, panel causality tests suggested by Toda and Yamamoto [52] (TY) and Dumitrescu and Hurlin [53] (DH) were performed. Both are modified versions of the causality test suggested by Granger [54]. According to Dumitrescu and Hurlin [53], the heterogeneous panel causality test is designed for bi-variates models of stationary and nonintegrated variables. The following DH non-causality equations were examined:

$$ \Delta  {HO}_{it}=\propto +\sum \limits_{k=1}^K{\beta}_{ik}\Delta \ {HO}_{i,t-k}+\sum \limits_{k=1}^K{\varnothing}_{ik}\Delta \ {HE}_{i,t-k}+{\in}_{i,t} $$

$$ \Delta  {HE}_{i,t}=\propto +\sum \limits_{k=1}^K{\beta}_{ik}\Delta \ {HE}_{i,t-k}+\sum \limits_{k=1}^K{\varnothing}_{ik}\Delta \ {HO}_{i,t-k}+{\in}_{i,t} $$

where *K* indicates the number of lag length in the balanced panel with ∝ is the intercept and slope coefficients are *β* and∅. As the test is sensitive to lag length [55], the formula *T > 5 + 2X* was used to determine the minimum number of lags where *X* signifies the minimum number of time needed at each number of lags and *T* is the time period [56]. The modified Wald (MWALD) causality test proposed by Toda and Yamamoto [52] is also used as it reduces the probability of inaccurate identification of the order of integration in the series by ignoring any possibility of non-stationarity and lack of cointegration in the panel [57, 58]. The modified Wald equation is:

$$ \Delta  {HO}_t={\alpha}_0+\sum \limits_{k=1}^n{\alpha}_{1k}\Delta \ {HO}_{t-k}+\sum \limits_{j=n+1}^{d_{max}}{\alpha}_{2j}\Delta \ {HO}_{t-j}+\sum \limits_{k=1}^n{\partial}_{1k}\Delta \ {HE}_{t-k}+\sum \limits_{\mathrm{j}=n+1}^{d_{max}}{\partial}_{2j}\Delta \ {HE}_{t-j}+{\omega}_{1t} $$

$$ \Delta  {HE}_t={\beta}_0+\sum \limits_{k=1}^n{\beta}_{1k}\Delta \ {HE}_{t-k}+\sum \limits_{j=n+1}^{d_{max}}{\beta}_{2j}\Delta \ {HE}_{t-j}+\sum \limits_{k=1}^n{\varnothing}_{1k}\Delta \ {HO}_{t-k}+\sum \limits_{j=n+1}^{d_{max}}{\varnothing}_{2j}\Delta \ {HO}_{t-j}+{\omega}_{2t} $$

The Toda-Yamamoto test increases the accurate order of the VAR system, *n*, precisely to the maximum order of integration, *d*_*max*_ [58]. Lastly, the approach uses seemingly unrelated regression (SUR) to estimate the model [59].

These two causality tests (DH and TY approach) are appropriate given the panel was heterogeneous, stationary at different levels (in some cases) and not cointegrated in the long-run for some of the models.

Finally, to measure the impact of an unexpected shock in the vector autoregressive (VAR) model, IRF and FEVD tests were performed. According to Swanson and Granger [60], IRF and FEVD tests proposed by Sims [61] are an integral part of the VAR estimations. The IRF examines the impact of shocks in cross-section by tracing the marginal effect of a shock to one variable in the system for the response variable [25]. On the contrary, FEVD shows the fraction of the changes in the dependent variable that is subject to their own shocks against the shocks to the impulse variable [62]**.** For further discussion on the methods of IRF and FEVD (Sims [61], Lütkepohl and Krätzig [63], and Brooks [62]).

Prior to these tests, the stability condition of the VAR was checked with the VAR stability test.

#### Diagnostic tests

To identify serial correlation in the panel data model the test developed by Wooldridge [31] is used. This test uses less assumptions, which are more robust and appropriate for micro panels. To examine the level of cross-section dependence (CD) in the panel data, the CD test of Pesaran [64] was performed. The test assumes zero mean for fixed values of *T* and *N,* and appropriate for heterogeneous, non-stationary and dynamic panel models [65]. In addition, modified Wald test for group wise heteroscedasticity and residual normality test [66] were conducted.

## Results

### Panel unit root tests

The results from cross-sectional augmented IPS (CIPS) and HT panel unit root tests for the level and first difference series are presented in Table 2. The CIPS heterogeneous panel unit root test indicated that HEPC, IMR, MMR, and U5MR are stationary at first difference. For higher income group, MMR, and for the lower-middle income group, U5MR is stationary at level. However, LFE is stationary at level and 1st difference for all income group.

**Table 2:** Panel unit root test results

Cross-sectional Augmented IPS (CIPS*) (Trend)										
Dep.Variables HE			IMR		MMR		U5MR		LFE	
Income Groups	Level	1st Diff	Level	1st Diff	Level	1st Diff	Level	1st Diff	Level	1st Diff
GL	−2.13	− 3.74*	− 2.42	− 3.31*	− 2.54	− 4.26*	− 2.65	− 2.88*	− 2.95*	− 3.83*
LY	− 2.70	− 4.27*	−2.38	− 2.72*	− 1.90	− 3.33*	−2.04	− 2.71*	−2.85*	− 2.77*
LM	−2.44	− 3.44*	− 2.53	−2.98*	− 2.09	− 3.16*	− 3.23*	3.86*	− 3.11*	− 3.98*
HM	− 2.52	− 3.54*	− 2.12	−2.95*	− 2.36	− 4.42*	−2.17	− 2.96*	− 3.98*	− 4.13*
HY	− 1.49	− 2.94*	−2.02	− 3.09*	2.75*	− 5.12*	− 2.21	− 3.02*	− 2.73*	− 4.42*
Harris–Tsavalis (HT) (Trend)										
Dep. Variables HE			IMR		MMR		U5MR		LFE	
Income Groups	Level	1st Diff	Level	1st Diff	Level	1st Diff	Level	1st Diff	Level	1st Diff
GL	5.51	−16.51*	8.52	− 5.42*	12.34	9.13^+^	− 12.27*	−41.78*	13.98	6.05^+^
LY	1.07	− 13.35*	0.61	− 14.90*	6.02	5.22^+^	−13.20*	− 27.22*	8.87	6.69^+^
LM	4.17	−12.82*	7.12	5.54^+^	5.71	−5.79*	6.09	−7.17*	12.12	10.27^+^
HM	2.97	−10.25*	5.34	3.62^+^	5.92	−3.88*	5.93	3.29	1.68	−4.33*
HY	3.23	−10.26*	6.99	−5.16*	− 0.66	− 15.58*	6.88	5.92	4.37	−7.24*
Cross-sectional Augmented IPS (CIPS*) (Constant)										
Dep. Variables HE			IMR		MMR		U5MR		LFE	
Income Groups	Level	1st Diff	Level	1st Diff	Level	1st Diff	Level	1st Diff	Level	1st Diff
GL	−2.20*	−3.69*	−2.47*	− 2.53*	−2.42*	−3.68*	− 2.78*	−2.45*	− 2.12	−3.17*
LY	−3.16*	−4.37*	−2.78*	− 2.45*	−1.36	−2.50*	− 2.39*	2.31*	− 3.33*	− 2.88*
LM	− 2.82*	−3.79*	− 2.44*	−2.32*	− 2.09	−3.17*	− 3.23*	−2.51*	−1.14	−3.10*
HM	−3.25*	−3.57*	−2.30*	--2.61*	−2.63*	−3.80*	−2.42*	−1.81	−1.87	−4.18*
HY	−1.83	−3.41*	−2.03*	−3.10*	−2.75*	−4.63*	− 2.32*	− 3.02*	−2.38*	− 4.23*
Harris–Tsavalis (HT) (Constant*)*										
Dep. Variables HE			IMR		MMR		U5MR		LFE	
Income Groups	Level	1st Diff	Level	1st Diff	Level	1st Diff	Level	1st Diff	Level	1st Diff
GL	11.05	−54.82*	10.16	−64.33*	9.35	−27.68*	6.72	−98.98*	12.27	−27.33*
LY	2.51	−16.51*	8.51	−5.41*	9.34	−27.68*	6.72	−98.97*	6.88	−30.25*
LM	4.17	−12.82*	7.21	−6.35*	3.83	−32.64*	5.27	−27.17*	7.08	1.13^+^
HM	2.87	−10.16*	5.27	3.57^+^	1.51	−5.46*	5.01	−7.91*	6.20	−34.49*
HY	11.05	−54.82*	10.16	−64.32*	9.34	−27.68*	6.72	−98.97*	11.00	−27.33*

Notes: *Significant at the 5% confidence interval. ^+^Not stationary at 1st difference. The optimal lag used for CIPS* test was based on MBIC and MAIC lag selection criteria. For the CIPS test the results of trend option are presented and the HT test the ‘Demean’ option was used to control for cross-sectional means. Here, *HE* Health expenditure per capita, *GL* Global, *LY* Low-income, *LM* Lower-middle, *HM* Higher-middle and *HY* High-income countries

The HT test showed similar results to CIPS in most of the cases except for the variable LFE. Interestingly, the test showed IMR for higher-middle and LFE for lower-middle income countries are not stationary either at level or at first difference. Based on these test results the ARDL (MG) test was applied to investigate the long-run association between the measures of IMR, MMR, U5MR and LFE with HE.

### Panel cointegration test

The ARDL test provides efficient estimates when the variables are cointegrated at dissimilar levels [44, 46] and report both short-run and long-run relationship between the variables. The findings in Table 3 indicate mixed results. It can be seen that IMR and LFE are cointegrated with HE in the short-run for all income levels. LFE also demonstrated long-run cointegration. However, MMR is cointegrated in the short run only for lower-middle and higher income countries and U5MR for lower-middle income countries. The long run coefficients showed a different picture. All the measures of HO have a long-run relationship with HE for low-income, higher-middle, and high-income countries.

**Table 3:** Mean group estimation ARDL model

Dep.Variables	IMR		MMR		U5MR		LFE	
Income Groups	*Long*	*Short*	*Long*	*Short*	*Long*	*Short*	*Long*	*Short*
LY	−0.22* (0.04)	− 0.01* (0.01)	0.48* (0.14)	− 0.11 (0.08)	0.03* (0.04)	− 0.03 (0.02)	− 0.01* (0.25)	− 0.21* (0.03)
LM	− 0.01 (0.03)	−0.04* (0.01)	− 0.02 (0.04)	−0.26* (0.09)	− 0.01 (0.04)	−0.07* (0.16)	0.013* (0.08)	−0.44* (0.05)
HM	0.124* (0.02)	−0.02* (0.07)	0.05* (0.28)	0.01 (0.08)	0.07* (0.04)	−0.03 (0.05)	0.04* (0.00)	− 0.51* (0.09)
HY	0.00* (0.00)	−0.08* (0.14)	−0.01* (0.00)	− 0.08* (0.05)	0.00* (0.00)	−0.09 (0.15)	0.00* (0.00)	−1.15* (0.11)

Notes: Figures in brackets are the standard errors. * Significant at 5% level. Lag order is chosen using AIC criterion. The estimated constant terms are significant at 5% confidence interval for all the model

To estimate the robustness of the findings of long-run relationship the FMOLS approach was employed. The results of the FMOLS test (Table 4) confirmed the long-run association between HO and HE. The only exception was MMR, which indicates no long-run cointegration with HE for lower and lower-middle income countries. Therefore, it is concluded that HO and HE were cointegrated in the long-run, however, the results vary based on the level of income and measures of HO used.

**Table 4:** Fully modified ordinary least square (FMOLS) test

Dep. Variables	IMR		MMR		U5MR		LFE	
Income Groups	*Coef.*	*SE*	*Coef.*	*SE*	*Coef.*	*SE*	*Coef.*	*SE*
GL	−0.003*	0.000	−0.024*	0.004	−0.004*	0.001	0.092*	0.006
LY	−0.005*	0.001	0.059	0.306	−0.080*	0.047	0.930*	0.180
LM	−0.025*	0.003	0.034	0.028	−0.040*	0.004	0.523*	0.085
HM	− 0.005*	0.001	−0.100*	0.017	−0.006*	0.002	0.209*	0.020
HY	−0.002*	0.001	− 0.036*	0.011	−0.000*	0.000	0.014*	0.003

Notes: * Indicates significant at 5% confidence interval

### Granger causality test

After confirming the long-run association, the VAR Granger panel causality test, the DH panel causality test and TY approach to Granger causality test were performed. The DH and TY approaches provide more reliable results as the panel data is heterogeneous, that is, variables are stationary at different levels and showed mixed results in the cointegration relationship.

In the DH causality test, HO and HE indicated bi-directional causality. Nevertheless, the results differ at different income levels. There are bidirectional relationships for IMR and U5MR with HE for low-income and lower-middle income countries. For high-income countries U5MR has bidirectional causality. The test found no causality for MMR with HE at any income level.

Again, the TY approach also illustrated bi-directional causality for IMR and U5MR with HE only for low-income countries. In addition, there is uni-directional relationship running from HE to IMR for high-income and lower-middle income countries. Thus, according to the TY causality test, HE Granger causes IMR and U5MR only for low-income countries.

The results in Table 5 indicated that the causal relation between HE and HO depends on income level, type of variables used to measure HO and choice of estimation techniques. Therefore, the results of the past studies that concluded a significant causal relationship should be interpreted with caution.

**Table 5:** Granger causality and non-causality tests

	Granger causality test		DH non-causality approach		TY non-causality approach	
	HE to IMR	IMR to HE	HE to IMR	IMR to HE	HE to IMR	IMR to HE
	*Z-value*	*Z-value*	*Z-value*	*Z-value*	*Z-value*	*Z-value*
GL	2.91	14.37*	9.29*	10.31*	6.69	7.32
LY	7.77*	0.43	3.19*	5.97*	4.57*	12.31*
LM	4.25	14.10*	3.02*	3.06*	5.79*	1.94
HM	2.88	1.92	1.54	1.65*	1.74	6.01
HY	22.57*	30.64*	4.02*	1.22	12.21*	5.79
	Granger causality test		DH non-causality approach		TY non-causality approach	
	HE to MMR	MMR to HE	HE to MMR	MMR to HE	HE to MMR	MMR to HE
	*Z-value*	*Z-value*	*Z-value*	*Z-value*	*Z-value*	*Z-value*
GL	5.76	10.67*	3.65*	4.01*	4.12	3.35
LY	0.20	1.07	−1.64	1.20	9.43*	1.95
LM	2.34	0.46	0.11	−0.84	5.29	5.89
HM	0.91	0.54	−0.24	− 0.12	5.39	0.01
HY	2.85*	0.56	−0.97	1.78*	1.87	6.37
	Granger causality test		DH non-causality approach		TY non-causality approach	
	HE to U5MR	U5MR to HE	HE to U5MR	U5MR to HE	HE to U5MR	U5MR to HE
	*Z-value*	*Z-value*	*Z-value*	*Z-value*	*Z-value*	*Z-value*
GL	7.97*	22.07*	9.26*	12.06*	5.52	5.02
LY	12.61*	0.55	4.51*	5.115*	10.19*	13.75*
LM	8.19*	1.49	4.36*	6.62*	1.67	1.65
HM	0.82	0.23	1.02	1.103	2.88	5.98
HY	35.58*	26.51*	3.82*	2.001*	3.27	3.44
	Granger causality test		DH non-causality approach		TY non-causality approach	
	HE to LFE	LFE to HE	HE to LFE	LFE to HE	HE to LFE	LFE to HE
	*Z-value*	*Z-value*	*Z-value*	*Z-value*	*Z-value*	*Z-value*
GL	11.85*	14.33*	16.30*	22.60*	52.21*	24.92*
LY	0.59	0.21	2.73*	31.17*	2.85	4.02
LM	2.30	0.28	2.18*	10.13*	5.2	14.04*
HM	1.32	0.56	1.78	37.38*	10.46	13.42*
HY	0.00	1.28	13.25*	19.62*	33.34*	5.21

Notes:* Indicates significant at 5% confidence interval. Lag lengths based on Modified AIC and Modified BIC lag selection criteria

### Diagnostic tests

Several diagnostic tests were performed to investigate key characteristics of the data. The diagnostic tests reveals that the model with LFE (dependent variable) and HE (independent variable) has first order serial correlation and the residuals are not normal. Therefore, no substantive conclusion about the relationship between LFE and HE can be made. Table 6 presents all the results of the diagnostic tests. The Wooldridge test for autocorrelation rejected the null hypothesis of first order autocorrelation for all models at all income levels except for the model of LFE and HE. The Pesaran CD test significantly accepts the alternative hypothesis of the presence of the cross-section dependence among the panel data. Moreover, the modified Wald test also found that the panel data contains heterogeneity. Lastly, the residual normality test on the panel data accepts the null hypothesis, therefore, it is concluded that the residuals of the panel data are normally distributed.

**Table 6:** Key diagnostic tests

	Wooldridge test for autocorrelation							
	HE -IMR		HE-MMR		HE –U5MR		HE-LFE	
	*Z-value*	*p-value*	*Z-value*	*p-value*	*Z-value*	*p-value*	*Z-value*	*p-value*
GL	2.54	*0.113*	0.84	*0.359*	3.76	*0.054*	27.97	*0.000*
LY	7.12	*0.067*	4.92	*0.073*	3.36	*0.075*	62.56	*0.000*
LM	1.39	*0.243*	1.46	*0.232*	5.16	*0.068*	1.13	*0.293*
HM	2.54	*0.112*	0.845	*0.359*	3.75	*0.054*	0.392	*0.532*
HY	3.74	*0.056*	0.092	*0.763*	12.76	*0.081*	15.89	*0.000*
	Pesaran CD test				Modified Wald heteroscedasticity test			
	HE to IMR HE-MMR		HE –U5MR HE-LFE		HE to IMR HE-MMR		HE –U5MR HE-LFE	
	*Z-value*	*Z-value*	*Z-value*	*Z-value*	*p-value*	*p-value*	*p-value*	*p-value*
GL	0.45*	0.25*	0.48*	0.86*	0.000	0.000	0.000	0.000
LY	0.45*	0.37*	0.43*	0.54	0.000	0.000	0.000	0.000
LM	0.54*	0.29*	0.56*	0.89*	0.000	0.000	0.000	0.000
HM	0.46*	0.23*	0.49*	0.26*	0.000	0.000	0.000	0.000
HY	0.41*	0.72*	0.45*	0.68*	0.000	0.000	0.000	0.000
Residual Normality test								
	Skewness	Kurtosis						
	*p-value*	*p-value*						
IMR- HE	0.273	0.167						
MMR-HE	0.095	0.205						
U5MR-HE	0.774	0.165						
LFE-HE	0.756	0.002						

Notes:* Indicates significant at 5% confidence interval

The null hypothesis for the: Wooldridge test is Ho: no serial correlation; Pesaran CD test is Ho: no cross-sectional dependence; MWALD heteroscedasticity test is Ho: Presence of homoscedasticity or constant variance. Residual normality test is Ho: Residuals are normally distributed

A vector autoregressive normality test (not reported) was also undertaken and all the eigenvalues indicated that the panel VAR is stable for all models.

### Impulse response and variance decomposition tests

**Fig. 1:**
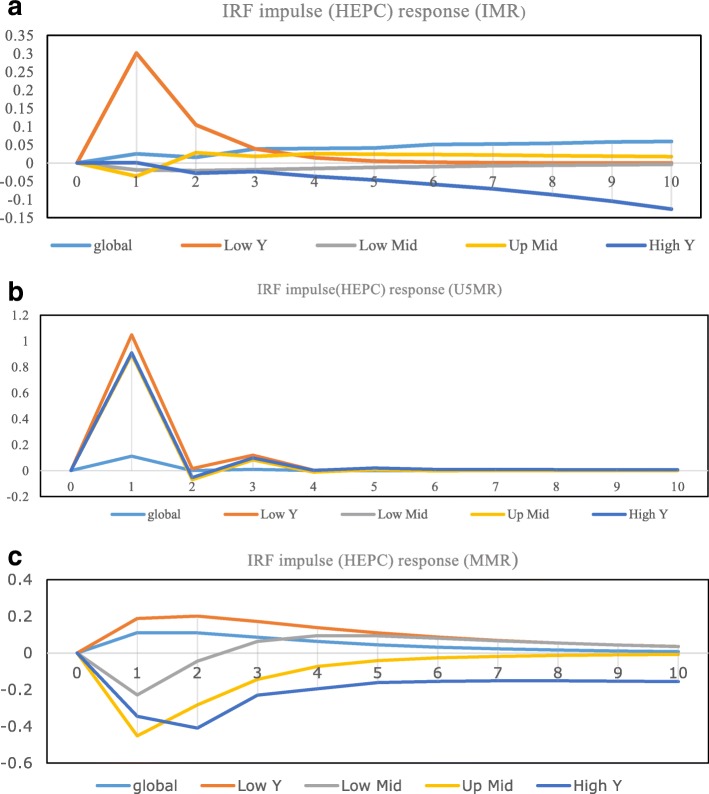
Impulse response functions. Health expenditures (impulse variable) and health outcomes (response variable). **a**) Title: IRF (impulse HEPC and response IMR). Legends . **b**) Title: IRF (impulse HEPC and response U5MR). Legends .  **c**) Title: IRF (impulse HEPC and response MMR). Legends

The Granger causality test suggested significant impact and direction of causality among the variables in the model but the results do not represent the construction or duration of these impacts. The results of the impulse responses of the variables are presented in Fig. 1 and variances decompositions in Table 7. The results of the IRF test indicated that any shock to HE per capita increases IMR and U5MR but the effects die down after two to three periods. Interestingly, MMR, except in low-income countries, reacted negatively to any shock to HE. Expectedly, the results showed that HO of low-income countries are more vulnerable to any shock in the model. Again, any shocks to HE have more impact on U5MR than the other measures of HO.

**Table 7:** Forecast-error variance decomposition test (income group comparison)

Variance decomposition			Impulse variable health expenditure				
LY	Response variables			LM	Response variables		
Period	*IMR*	*MMR*	*U5MR*	Period	*IMR*	*MMR*	*U5MR*
0	0	0	0	0	0	0	0
2	0.1516875	0.0168	0.083523	2	0.019212	0.006631	0.062427
4	0.1717245	0.0408	0.083659	4	0.022824	0.016638	0.06754
6	0.172093	0.0508	0.083663	6	0.02724	0.020663	0.068715
8	0.1720995	0.005505	0.083663	8	0.030114	0.022065	0.068956
10	0.1721	0.0057	0.083663	10	0.031903	0.02254	0.069006
12	0.1721	0.005795	0.083663	12	0.033052	0.0227	0.069017
HM	*Response variables*			HY	*Response variables*		
Period	*IMR*	*MMR*	*U5MR*	Period	*IMR*	*MMR*	*U5MR*
0	0	0	0	0	0	0	0
2	0.0094175	0.008848	0.014273	2	1.69E-05	0.003191	0.12657
4	0.083379	0.011133	0.046812	2	0.041963	0.019582	0.161816
6	0.102849	0.011687	0.057516	2	0.061122	0.030531	0.213393
8	0.1067585	0.01194	0.062043	8	0.074016	0.047492	0.227842
10	0.1075825	0.012068	0.064321	10	0.082359	0.061703	0.232503
12	0.1077575	0.012136	0.065539	12	0.087124	0.07372	0.23381

Here, *GL* Global, *LY* Low-income, *LM* Lower-middle, *HM* Higher-middle and *HY* High-income countries

The results in Table 7 are consistent with the findings of the IRFs and reveal that HE can explain future variations in U5MR more than any other measures of HO at all income levels. HE can explain as much as 17.21% of the variation in IMR after the 12th year for low-income countries. Results of the FEVD test showed that the relationship between MMR and HE is insignificant at all income levels.

### Robustness to alternative specifications

To examine the robustness of the findings, this study estimated two alternative hypotheses by adding a variable to the model and by identifying the impact of a common shock on the relationship. First, the ARDL and FMOLS tests were conducted by incorporating total factor productivity (TFP) into the model. Previous studies have concluded that improvement in TFP has a positive and significant influence on HO [67, 68]. A higher level of TFP indicates the efficiency with which resources are utilised [69]. Hence, countries with higher TFP often produce better HO with lower spending on health. The results (Tables 8 and 9 in Appendix) showed no significant difference in the association between HE and HO when TFP was controlled for except for the variable MMR. HE and MMR demonstrated a significant and negative relationship for lower, lower-middle and higher-middle income countries. For detailed definition of TFP and the source of data see the ‘Conference Board Total Economy Database’ [70].

Second, previous studies have concluded that the global financial crisis (GFC) of 2008 had a severe impact on health, health-related behaviours and quality of life. There is evidence to support this link for both developed countries [71, 72] as well as for developing countries [73, 74]. These studies associated the GFC with a reduction in health funding and subsequent poor HO in the post GFC period. Hence, this study further investigated the long-run relationship between HE and HO by dividing the panel data into pre (1995–2008) and post (2009–2014) GFC periods. The outcomes of panel ARDL model and the FMOLS estimations for the two periods illustrate minor differences in the long-run relationship between HE and HO for middle-income countries (Tables 10 and 11 in Appendix). In particular, for the lower-middle and higher-middle income countries the IMR and U5MR show substantial long-run association with HE in the post GFC period only.

## Discussion

The findings indicated that the causal relationship varies significantly between low, middle and high-income countries. Several major discussion points arise.

First, the causal link between HE with IMR and U5MR is greater for low-income countries. Although there is evidence of a significant association at all income levels, the results are more consistent for low-income countries across all the measures of HO. These findings are similar to Self and Grabowski [17] and Deaton [75] who concluded that rising HE has greater influence on HO in low-income countries than in higher income countries. In addition, high-income countries have better HO because they continuously enjoy better health over a longer period of time. As IMR is already at minimum levels in these countries, rising HE has no significant influence on its further reductions.

Second, HE has no causal relation with MMR. Therefore, countries need to focus on other unobserved variables to make a substantial impact in its reduction. Gottret and Schieber [76] and Nicholas et al. [19] also reached similar conclusions. Compared to child mortality which is an outcome of primary care, MMR is viewed as secondary care which is often provided by hospitals [23]. In low-income countries, especially in rural areas, the lack of infrastructure and distance to the nearest hospital reduce access to health care services. Wagstaff [77] found that the quality of the road network can influence the impact of HE on HO. In addition, it is accepted that a lack of family planning and minimum access to health care are the major causes of MMR [17]. Again, a closer look at the data reveals that MMR in high-income countries is already very low, averaging 10 deaths per 100,000 live births. Subsequently, further spending may not induce any significant reduction in MMR. A further analysis of the relationship indicated that the relationship became significant for all income groups except high-income countries once TFP is accounted for in the model.

Third, ARDL and FMOLS test results indicated that HE has a positive impact on LFE in the short-run as well as in the long-run. Similar findings are evident from [14, 21]. Noticeably, the impact of HE on LFE is higher in lower income countries compared to higher income countries with better LFE. Therefore, the marginal return of HE on LFE diminishes as the LFE grows higher.

Lastly, any negative economic shock in HE affects HO in the low and lower-middle income countries to a proportionally greater extent. That means, poor countries are more exposed to negative shocks. In addition, HE can explain variations in IMR and U5MR more than the other two measures of HO.

The variations in the relationships between HE and HO among income groups may be subject to differences in the health financing mix and level of efficiency in allocating scarce resources and providing health care services [78]. According to Self and Grabowski [17] developed countries often enjoy the virtuous cycle of good health because of higher incomes and levels of education. Previous studies have also indicated that efficient health interventions and ease of access to health care services often play a moderating role in shaping the effectiveness of the health care expenditure [14, 77]. Unfortunately, these measures vary significantly among countries of different income groups.

In addition, heterogeneity in the HO of the population often results from lifestyle choices (consumption of alcohol and tobacco) [79]; obesity and other chronic diseases [80]; inequality in income distribution; level of female education; ethnic diversity, and religious beliefs [14]. Others have concluded that the magnitude of the association between HE and HO depends on the availability and allocation of medical resources [81, 82] and the efficiency with which these resources are utilized [83]. For lower and middle-income countries, increases in HE on immunization and vaccination programs can substantially reduce child mortality [29, 84]. Another key issue is to realize that there is evidence of diminishing marginal returns to growing HE in the health care sector for developed countries [85]. This indicates that identical amounts of HE in lower and middle-income countries would generate higher HO compared to higher income or developed countries [5]. Hence, the findings in this current study of significant heterogeneity in the relationship between HE and HO across income levels is justified.

Lastly, the results depend on the choice of assumptions made including, homogeneity, cross-section independence, serial correlation and residual normality. Again, the findings vary substantially on the selection of lag values, therefore, like other macroeconomic variables, measures of HO also depend on their values in previous years. Moreover, inappropriate diagnostic tests may lead to incorrect selection of estimation techniques and spurious results.

Some important policy implications can be drawn from the findings of the study. Firstly, the marginal impact of additional per capita health expenditure decreases as the level of expenditure increases. This is evident from the empirical results as a dollar increase in health expenditure in low-income countries demonstrates a greater increase in health outcomes, compared to higher income countries. Another important consideration is that mortality rates in developed countries are considerably lower consistent with a higher level of life expectancy. Therefore, these countries need to allocate additional funds (much larger than low or middle-income countries) for further improvement in health outcome as their population ages. Secondly, lower income countries are more adversely affected by negative shocks in the health care sector. Policymakers in these countries should maintain additional provisions for these shocks (disease or finance). Else, the health outcome achievements of one decade might be dissipated within a very short period of time. Thirdly, the descriptive analysis showed that countries of all income levels have experienced lower mortality and rising life expectancy during the last two decades. This indicates that low and middle-income countries will soon experience a change in their demographic structure. Existing financial mechanisms might be inadequate to support the additional demand for medical services that will arise. Lastly, lack of adequate information on health, especially in the low-income countries often makes it difficult to conduct empirical analysis and draw accurate conclusions. Governments in each country should devote resources towards high quality health data collection and make it available for further empirical investigation.

Future empirical studies should focus on analysing the relationship between health outcomes and expenditure, using country-specific data. Specifically, understanding the mediating role of good governance, level of infrastructure development, productivity, economic development and health financing mix to explain the impact of health expenditure on health outcomes.

This study also has some limitations. First, due to data unavailability the time period studied is relatively short at 20 years; second, the unavailability of a comparable health index as proxy for HO; third, an accurate causality test on the relationship between LFE and HE could not be made as the data showed autocorrelation; fourth, the exclusion of key determinants of HO; and finally, no control for country specific key factors influencing HO at different levels of income was undertaken. Moreover, from the methodological perspective, this study attempted to address some of the common problems associated with panel cointegration and panel VAR Granger causality tests. For instance, the issues of stationarity and heterogeneity of the data and cross-section dependence have been accounted for with appropriate estimation techniques using a strongly balanced panel data. Nonetheless, the results of this study should be interpreted with caution due to some underlying assumptions of the estimation techniques. For example, the Toda-Yamamoto test fails to distinguish between short-run and long-run causality [86] and the cross-section panel data does not take into account the country-specific characteristics (population, geography, governance and productivity) [87]. Due to the bivariate causality model, there may be the possibility of omitted variable bias. These issues are for future empirical research.

Lastly, this study did not account for measurement errors in the dependent and explanatory variables. Despite the ability of panel data to account for measurement errors [88], ignoring the problem especially in the explanatory variables might generate biased and inconsistent OLS estimates [31, 37, 89]. Gujarati [90] stated that a perfect solution to measurement errors in the panel data is unavailable however, there are some suggestions in the literature to account for the problem in the econometric analysis with an instrumental variable approach [89] and ‘Generalised Methods of Moments’ estimation [31, 37]. Therefore, future studies may use these methods to examine the association between HE and HO. Any variations in findings may prove the presence of measurement error in the HE data produced by the World Bank.

## Conclusions

This study examined the relationship between HO and HE for 161 countries for the period of 1995 to 2014. The findings from the panel cointegration test reveal that the HO measures are significantly associated with HE both in the short and long run and across all income levels. However, the short-run relation is stronger. More importantly, the heterogeneous panel DH causality test indicated that IMR and UM5R have bidirectional relationships with HE for all income levels except higher middle-income countries. However, according to the TY causality approach, HE Granger causes HO only for the low-income countries. This indicates that income does play an important moderating role in determining how much HE influences HO. However, the findings vary significantly due to the choice of estimation techniques.

In addition, lower income countries are more at risk of adverse HO because of negative shocks to HE. Variations in IMR and U5MR are better explained by HE than MMR. Lastly, the diagnostic test results vary significantly due to the choice of assumptions and lag order selection.

It is evident from the findings that increasing HE alone will not generate maximum HO at any income level. The policy makers need to look into the mix of HE and the allocative efficiency of the utilised resources [12, 78]. Therefore, countries have the potential to achieve better HO through an efficient composition of HE and its financing mix. For example, rising HE has an insignificant impact on reducing MMR. Therefore, for better outcomes policy makers should focus on effective interventions such as family planning, increasing productivity of the health care sector and access to affordable health care. Again, theoretical and empirical research is needed to analyse the composition of HE and to build a comparable measure of HO for all countries. Improved/better HO measures like quality adjusted life years and potential years of life lost should be widely available.

## Appendix A

**Table 8:** Mean group estimation ARDL model (with total factor productivity)

Income Groups	Independent variables	IMR		MMR		U5MR	
		*Long*	*Short*	*Long*	*Short*	*Long*	*Short*
LY	HEPC	0.49* (.15)	−0.03*(.004)	1.7(2.6)	−0.25*(.10)	−0.42*(.10)	−0.10*(.00)
*N* = 24	TFP	−0.52 (.41)	0.003(.02)	1.2*(5.1)	−0.21(.15)	−4.1*(.38)	0.01*(.03)
LM	HEPC	−0.003*(.00)	−0.05*(.01)	− 0.01*(.00)	− 0.01(0.01)	− 0.01(.00)	− 0.005(.02)
*N* = 32	TFP	0.04(.02)	0.00(.002)	0.21*(.03)	− 0.13(0.07)	0.01(.02)	0.002(.01)
HM	HEPC	0.01 (.001)	− 0.01(.01)	− 0.01*(.00)	− 0.002(.00)	0.01*(.00)	− 0.002(.01)
*N* = 22	TFP	−0.18*(.07)	0.01*(.003)	0.15(.12)	−0.05(.02)	−0.02(.06)	0.01(.01)
HY	HEPC	−0.002*(.00)	−0.001*(.01)	− 0.003*(.00)	−0.003*(.00)	− 0.002*(.00)	−0.001*(.00)
*N* = 37	TFP	−0.01(.02)	−0.002(.001)	0.02(.03)	−0.02(.01)	− 0.02(0.01)	−0.001(.001)

Notes: Figures in brackets are the standard errors. * Significant at 5% confidence interval. N indicates number of countries in each income groups

**Table 9:** Fully modified ordinary least squares test (with total factor productivity)

Income groups	Independent variables	IMR		MMR		U5MR		LFE	
		Coef.	SE	Coef.	SE	Coef.	SE	Coef.	SE
LY	HEPC	−0.02	0.03	−0.59*	0.29	0.24*	0.06	0.04*	0.01
	TFP	0.77*	0.03	−1.12*	0.04	−0.32*	0.08	0.74*	0.02
LM	HEPC	−0.03*	0.00	0.01	0.04	−0.04*	0.01	0.02*	0.002
	TFP	0.18*	0.02	0.29*	0.02	0.46*	0.03	0.18*	0.009
HM	HEPC	−0.01*	0.00	0.03*	0.003	−0.02*	0.00	−0.003	0.001
	TFP	−0.16*	0.12	0.15*	0.06	−0.17*	0.02	−0.14	0.014
HY	HEPC	−0.00	0.00	0.001*	0.00	−0.00*	0.00	0.003	0.000
	TFP	−0.18*	0.00	−0.30*	0.01	−0.22*	0.01	−0.59	0.012

Notes: * Indicates significant at 5% confidence interval. Coef. indicates the coefficients and SE is the standard error. Lag orders were chosen using AIC criterion

**Table 10:** Mean group estimation ARDL model

(Pre GFC, Year = 1995 to 2008)					
Income Groups		IMR		U5MR	
		*Long*	*Short*	*Long*	*Short*
LY	HEPC	−0.14* (.02)	−0.29* (.14)	− 0.14* (.02)	−0.03*(.01)
LM	HEPC	−0.00 (.00)	−0.06* (.02)	− 0.00 (.00)	- 0.06* (.02)
HM	HEPC	−0.001 (.00)	−0.04* (.01)	−.001 (.00)	− 0.05* (.01)
HY	HEPC	−0.001* (.00)	−0.12* (.01)	− 0.00* (.00)	−0.12* (.02)
(Post GFC, Year = 2009 to 2014)					
Income Groups		IMR		U5MR	
		*Long*	*Short*	*Long*	*Short*
LY	HEPC	−0.03*(.00)	−0.13*(0.06)	−0.01 (.005)	− 0.14*(.06)
LM	HEPC	−.004* (.00)	−0.03 (.02)	−0.01* (.00)	− 0.08* (.03)
HM	HEPC	−0.00* (0.00)	−015*(.05)	− 0.001*(.00)	−0.11* (.05)
HY	HEPC	−0.00* (.00)	−0.04* (.02)	− 0.02* (.00)	−0.16*(.08) 9(.04)(0.04)

Notes: Figures in brackets are the standard errors. * Significant at 5% confidence interval

**Table 11:** Fully modified ordinary least squares test

(Pre GFC, Year = 1995 to 2008)					
Income Groups		IMR		U5MR	
		*Coef.*	*SE*	*Coef.*	*SE*
LY	HEPC	−0.20*	0.02	−0.26*	0.05
LM	HEPC	−0.04*	0.00	0.05*	0.01
HM	HEPC	−0.004*	0.00	0.00	0.00
HY	HEPC	−0.001*	0.00	0.00*	0.00
(Post GFC, Year = 2009 to 2014)					
Income Groups		IMR		U5MR	
		*Coef.*	*SE*	*Coef.*	*SE*
LY	HEPC	−0.06*	0.01	−0.19*	0.03
LM	HEPC	−0.01*	0.00	−0.01*	0.00
HM	HEPC	−0.004*	0.00	−0.02	0.00
HY	HEPC	−0.00*	0.00	−0.002*	0.00

Notes: Coef. indicates the coefficients and SE is the standard error. * Significant at 5% confidence interval. Lag order is chosen using AIC criterion

### List of Countries included in the study

The countries in this study have been divided into four groups according to the World Bank income group classifications. However, it is important to note that during this twenty year period (1995–2014) many countries improved their status from low-income to a comparatively higher-income country. For simplification of the study a country is considered a low-income country if for the majority of the time period it was a low income country. For instance, Ghana is currently a lower-middle income country. But in this study Ghana is considered a low income country as it was a low income country until the year 2009. The classification of the countries are given below:

**Table:**

1. High:	Antigua and Barbuda, Australia, Austria, The Bahamas, Bahrain, Barbados, Belgium, Brunei Darussalam, Canada, Cyprus, Czech Republic, Denmark, Finland, France, Germany, Greece, Iceland, Ireland, Israel, Italy, Japan, Rep. Korea, Kuwait, Luxembourg, Malta, Netherlands, New Zealand, Norway, Portugal, Qatar, Saudi Arabia, Singapore, Slovenia, Spain, Sweden, Switzerland, Trinidad and Tobago, United Arab Emirates, United Kingdom, United States.
2. Upper-middle:	Argentina, Belarus, Belize, Botswana, Brazil, Bulgaria, Chile, Costa Rica, Croatia, Cuba, Equatorial Guinea, Estonia, Gabon, Grenada, Hungary, Latvia, Lebanon, Lithuania, Malaysia, Mauritius, Mexico, Oman, Panama, Poland, Romania, Russian Federation, Slovak Republic, South Africa, St. Lucia, Turkey, Uruguay, Venezuela,.
3. Lower-middle:	Albania, Algeria, Angola, Armenia, Azerbaijan, Bhutan, Bolivia, Bosnia and Herzegovina, Cabo Verde, Cameroon, China, Colombia, Rep. Congo, Djibouti, Dominican Republic, Ecuador, Arab Rep. Egypt, El Salvador, Fiji, Guatemala, Guyana, Honduras, Indonesia, Islamic Rep. Iran, Jamaica, Jordan, Kazakhstan, Kiribati, FYR Macedonia, Maldives, Moldova, Mongolia, Montenegro, Morocco, Namibia, Nicaragua, Papua New Guinea, Paraguay, Peru, Philippines, Samoa, Sri Lanka, Swaziland, Thailand, Tonga, Tunisia, Turkmenistan, Ukraine, Uzbekistan, Vietnam.
4. Low:	Bangladesh, Benin, Burkina Faso, Burundi, Cambodia, Chad, Comoros, Dem. Rep. Congo, Cote d’Ivoire, Eritrea, Ethiopia, The Gambia, Ghana, Georgia, Guinea, Haiti, India, Kenya, Kyrgyz Republic, Madagascar, Malawi, Mali, Mauritania, Mozambique, Myanmar, Nepal, Niger, Nigeria, Pakistan, Rwanda, Senegal, Sierra Leone, Sudan, Tajikistan, Tanzania, Togo, Uganda, Rep. Yemen, Zambia.

## Abbreviations

AIC: Akaike information criterion
ARDL: Autoregressive distributed lag
BIC: Bayesian information criterion
CD: Cross-section dependence
DH: Dumitrescu & Hurlin
DY: Disposable income
FEVD: Forecast-error variance decomposition
FMOLS: Fully modified ordinary least squares
GFC: Global financial crisis
HE: Health expenditure
HEPC: Health expenditure per capita
HI: Health inputs
HO: Health outcomes
HT: Harris-Tsavails
IMR: Infant mortality rate
IPS: Im-Pesaran-Shin
IRF: Impulse response function
LFE: Life expectancy at birth
MG: Mean group
MMR: Maternal mortality rate
MWALD: Modified Wald
OECD: Organisation for economic co-operation and development
SUR: Seemingly unrelated regression
TFP: Total factor productivity
TY: Toda-Yamamoto
U5MR: Under-five mortality rate
VAR: Vector Autoregressive
